# Specimen Fasciculus of the Catalogue of the National Medical Library

**Published:** 1876-08

**Authors:** 


*Specimen Fasciculus of a Catalogue of the National Medical Library.* Under the direction of the Surgeon-General, United States Army, at Washington, D. C. Government Printing Office, 1876.

The National Medical Library contains about forty thousand volumes properly so called, and about an equal number of single pamphlets. To make this vast amount of material available, Assistant Surgeon J. S. Billings, in charge of the library, has prepared a catalogue, which is nearly ready for the press. The specimen which is here presented, has been prepared with a view of obtaining advice as to the form best suited to a catalogue of this character.

The specimen fasciculus exhibits the plan proposed and carries the catalogue as far as the letters A-I-R. The titles of monographs are arranged first, coming in the alphabetical order of their authors, for instance, under abdomen (abscess of) we have works by Eydam, Frabicius, Henricus, etc., in order of the alphabet. Following the enumeration of monographs are given the titles of articles in Medical Journals referring to the topic. As an illustration of the character of the work, we will refer to the term Abortion. Under this head are given the titles of one hundred and fifty-three special works, referring to the subject, together with the title and locality of over one hundred and seventy articles in medical journals upon the same topic. A division is also made of this same subject under the head of Jurisprudence of Abortion, under which title seventeen monographs are catalogued, and the titles of seventy-seven articles in medical journals enumerated.

The plan adopted by Dr. Billings, seems to us as perfect as any that can be suggested. In one portion of the work the names of authors are printed in italics, we think the plan of using small capitals, as employed after the conclusion of the subject abscess, preferable.

It is to be hoped that Congress will soon be made to see the desirability of ordering the catalogue printed. It is estimated than when complete it will make about five volumes of small quarto size, of one thousand pages each.

